# COVID-19 vaccination hesitancy among Malawians: a scoping review

**DOI:** 10.1186/s13643-024-02499-z

**Published:** 2024-02-28

**Authors:** Ellen Nkambule, Balwani Chingatichifwe Mbakaya

**Affiliations:** 1 Department of Nursing and Midwifery, Mzuzu University, Mzuzu, Malawi; 2 Department of Public Health, University of Livingstonia, Mzuzu, Malawi; 3 Department of Biological Sciences, Mzuzu University, Mzuzu, Malawi

**Keywords:** COVID-19, COVID-19 vaccine hesitancy, Scoping review, Malawi

## Abstract

**Background:**

The best chance of eradicating the COVID-19 pandemic lies in a successful vaccination campaign against the virus. There is still hesitancy among some of the Malawians over the use of COVID-19 vaccines that are readily available. This review’s objective was to currently analyze COVID-19 vaccination hesitancy among Malawians.

**Methods:**

This scoping review was conducted following the Preferred Reporting Items for Systematic Reviews and Meta-Analyses (PRISMA) extension for Scoping Reviews. An electronic database search was performed using CINAHL, OVID Medline, PubMed, and Google Scholar for studies published between January 1, 2020, and July 10, 2023, on the topic of reluctance toward COVID-19 vaccine in Malawi. A review of the journal titles and abstracts was performed to establish a match within the selection criteria. Based on the parameters of interest, this paper included publications that explicitly mentioned COVID-19 vaccine hesitancy in Malawi.

**Results:**

A total of seven articles were identified as meeting the inclusion criteria. Some of the eligible Malawian population’s poor response to COVID-19 vaccination was due to hesitancy. Misinformation is primarily to blame for COVID-19 vaccine reluctance. COVID-19 vaccines are viewed as dangerous or intended to cause harm such as the myth that the COVID-19 vaccine would cause infertility, severe disability, and even death. The review revealed that some people choose not to receive vaccines due to religious convictions and beliefs. Some individuals also think that getting the COVID-19 vaccine could result in receiving a triple-six (666) mark from the devil. There were also reports that the COVID-19 vaccination is being considered experimental and ineffective.

**Conclusions:**

The Malawi government should focus on fear and misinformation regarding COVID-19 vaccination campaigns, using interventions, motivational interviews, and individual sensitization. Traditional, religious, and youth-led organizations should provide practical information on COVID-19 vaccine safety and efficacy.

**Supplementary Information:**

The online version contains supplementary material available at 10.1186/s13643-024-02499-z.

## Introduction

Vaccination against the novel coronavirus (COVID-19) is the best solution for eradicating the virus [1, 2]. However, COVID-19 vaccination hesitancy has been documented in many parts of the world including sub-Saharan Africa (SSA) [2]. According to the World Health Organization (WHO), vaccine hesitancy is described as reluctance or delay in receiving vaccines regardless of the availability of immunization services [3]. The WHO [3] identified vaccine hesitancy as one of the top ten worldwide public health hazards in 2019. It is driven by a variety of reasons such as safety concerns, rumors and conspiracy theories, and fear of catastrophic events [3]. Currently, 26.8% of the SSA population is fully vaccinated against COVID-19, compared to more than 50% in the Global North [4].

The COVID-19 pandemic has had a remarkable global impact. Besides loss of life, the pandemic response had a negative impact on world health, education, trade, agriculture, and socioeconomic growth [5]. There is evidence that if the COVID-19 vaccine had been available at the start of the pandemic in 2019, we would not have endured lengthy lockdowns, a downturn in the economy, and a decline in mental health [6]. In addition, fewer individuals would have died [6]. The COVID-19 vaccine is currently viewed as a means to fight COVID-19. However, there are challenges as regards to the vaccine such as vaccine inequity, adverse side effects, and hesitancy. The Malawi government launched an action plan to increase the number of areas where people can get COVID-19 vaccines conveniently such as workplaces and shopping centers. However, there is still reluctance in Malawi about using easily available vaccines [7]. In March 2021, the government received a donation of 360,000 doses of AstraZeneca COVID vaccination as a first dose for eligible people [8]. The government prioritized this first shipment for people who were assessed to be at high risk of developing the COVID-19 pandemic such as healthcare workers, the elderly over 60 years, and individuals with underlying comorbidities like diabetes and asthma. The vaccine uptake was so low that 19,610 doses expired and were discarded in May 2021 [9]. While the rest of the world was attempting to combat COVID-19 through vaccination, most Malawians particularly those living in rural regions refused to get vaccinated. According to reports, COVID-19 vaccination was introduced in Malawi without sensitizing communities or conducting workshops [10]. Only 1,072,229 persons in Malawi received at least one dose of the AstraZeneca vaccine by December 26, 2021, out of a nationwide target population of 18,546,324; and only 672,819 had been fully vaccinated [11]. As of February 2023, just two million people (10.3%) of the target population of 13.8 million had been fully vaccinated [12]. Vaccine hesitancy remains a significant component of vaccine rollout that the Malawi government should address. Furthermore, vaccine hesitancy has been observed in a few surveys, although the prevalence of this problem in LMIC has yet to be thoroughly researched [13–17]. To inform steps to promote public acceptability and uptake of COVID-19 vaccinations, it is critical to understand the factors that impact COVID-19 vaccine hesitancy. This scoping review, therefore, aims at analyzing COVID-19 vaccination hesitancy among Malawians.

## Methods

This scoping review was conducted on 10th July 2023 following the Preferred Reporting Items for Systematic Reviews and Meta-Analyses (PRISMA) extension for Scoping Reviews [18]. This study aimed to answer the following research question: “what is influencing COVID-19 vaccine hesitancy in Malawi?” An electronic database search was performed using CINAHL, OVID Medline, PubMed, and Google Scholar on the topic of reluctance toward COVID-19 vaccine in Malawi. A review of the journal titles and abstracts was performed to establish a match within the selection criteria. Based on the parameters of interest, this paper included publications that explicitly mentioned COVID-19 vaccine hesitancy in Malawi. The publications that fit the inclusion criteria were obtained for a full-text review. PRISMA [18] propose a two-stage screening procedure, known as sequential or staged. In stage one, the articles were screened based on the title and abstract, and only those that passed this stage had their full content extracted. According to PRISMA, a second screening occurred based on the full text, with “Reports Assessed for Eligibility.” This struck a good compromise between quality and quantity, as the amount of first papers discovered during a systematic review can be alarming. Zotero software assisted in reference reduplication isolation and Microsoft Excel software was employed for the screening process. The review excluded publications that reported any form of vaccine hesitancy not related to COVID-19. To synthesize the review results, we qualitatively summarized the information and employed a data analysis approach to extract significant details from the papers included in this study.

### Eligibility criteria

Eligible studies were (1) peer-reviewed, published, and indexed in CINAHL, PubMed, OVID Medline, and Google Scholar; (2) primarily discussing or evaluating COVID-19 vaccine acceptance/hesitancy; (3) focused on Malawi; (4) published in English; and (5) published between January 1, 2020, and July 10, 2023. Letters to the editor, non-empirical research, reviews, or protocols, conference proceedings, reports, opinion pieces, and comments were all excluded.

### Search strategy

The searches on all four databases were done on 10th July 2023. Detailed search strategies and search results are presented in Additional file 1. Bibliographies of articles that were included for review were also scanned to capture any literature that was missed from the formal search. The phrase “Malawi” was used to find studies conducted in this nation. We did not incorporate a date filter because we expected papers on COVID-19 to be released during the epidemic.

### Data extraction

E.N. and B.C.M. created a data extraction form and independently conducted data extraction on the selected articles following the inclusion and exclusion criteria. The following information was extracted from the articles that were included for data extraction: last name of the first author, title, study design, and study aim.

## Results

**Fig. 1:**
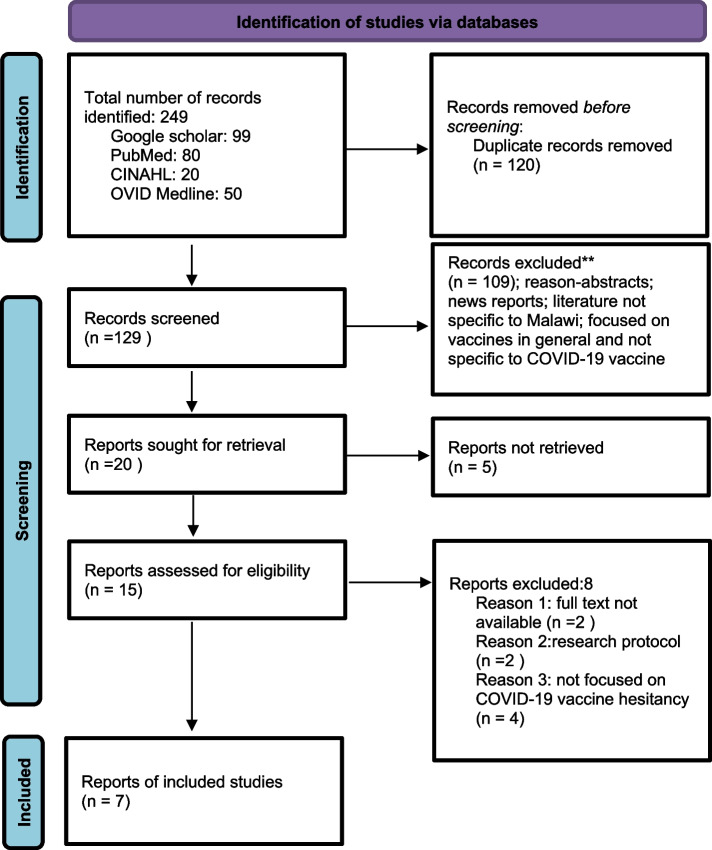
PRISMA flow chart to show the study selection process

From our initial search in the aforementioned databases, a total of 249 records were qualified for title and abstract screening. After removing duplicates (*n* = 120), 129 studies were eligible for the title and abstract screening. One hundred and nine (109) papers were deemed irrelevant and were eliminated; a good number of these researches focused on other African nations rather than Malawi and discussed vaccine reluctance in general rather than COVID-19 vaccine hesitancy in particular, leaving 20 studies for full-text screening. Five reports were not obtained during a full-text screening, leaving out 15 papers. Then, eight (8) studies were eliminated because they were protocols, did not focus on COVID-19 vaccine reluctance, or did not have the entire text available. The final analysis included the remaining seven papers (Table 1). The selection process is shown in the PRISMA flow diagram (Fig. 1).

**Table 1:** Table showing the eligible papers for analysis of vaccine hesitancy *n* = 7

Article	Title	Study design	Study aim
Chimatiro et al. [19]	Barriers affecting COVID-19 vaccination in Phalombe District, Malawi: a qualitative study	Cross-sectional qualitative study employing six focus group discussions (FGDs) and 19 in-depth interviews (IDIs) to collect data	The paper looks into reasons for vaccine refusal and hesitancy, how contextual cultural beliefs influenced people’s decision to receive the COVID-19 vaccine, and which sources of information were trusted in the community
Safary and Mtaita [20]	A qualitative exploration of perceptions of the COVID-19 vaccine in Malawi during the vaccine rollout phase	Online survey to collect free-text responses to assess factors leading to hesitation or refusal of COVID-19 vaccination in Malawi	The paper looks into factors leading to hesitation and/or refusal to COVID-19 vaccination in Malawi
Ao et al. [21]	Acceptance of COVID-19 vaccines among adults in Lilongwe, Malawi: a cross-sectional study based on the health belief model	A cross-sectional design was used for this field-based survey	The purpose of this study was to investigate current vaccination rates for COVID-19 among Malawians, assess the level of COVID-19 vaccine hesitancy among Malawians, and explore the factors influencing vaccination and willingness to be vaccinated against COVID-19
Aron et al. [22]	Attitudes toward COVID-19 vaccines among patients with complex non-communicable disease and their caregivers in rural Malawi	This cross-sectional study was nested in a prospective open cohort study among patients with complex NCDs in Neno	The paper advocates for strong trust in health care workers and community engagement that could help increase vaccine acceptance
Ojong and Agbe [23]	“This is most likely not the correct vaccine”: analyzing COVID-19’s viral spread and vaccine anxieties in Ghana, Cameroon, and Malawi	This paper uses a qualitative research approach that included 144 semi-structured interviews	The focus of this paper is on vaccine anxiety, which is a significantly understudied area in the public health discourse. Specifically, the authors examined how social and political dynamics shaped people’s perspectives in particular environments in Ghana, Cameroon, and Malawi about COVID-19 vaccines and the spread of COVID-19
Holden et al. [24]	Religion, beliefs, trust, and COVID vaccination behavior among rural people in Malawi	Quantitative research approach	This study investigated the religious and other beliefs related to the corona/COVID-19 pandemic and how they are related to COVID-19 risk perceptions, trust in COVID vaccines, and how these are affected by the religious beliefs, religious affiliations, trust in authorities, generalized trust, and how these affect vaccine demand/vaccine hesitancy
Mchawa et al. [25]	Assessing SARS-CoV-2 vaccine hesitancy among the people living with and without HIV from May to September 2022 in Blantyre, Malawi	Cross-sectional study	To find out the status of SARS-CoV-2 vaccine hesitancy among PLHIV and explore the influencing factors from May to September 2022 in Blantyre, Malawi, and then put forward feasible measures to improve the situation based on the findings

### Factors related with/reasons for COVID-19 vaccine hesitancy

The results of the reviewed papers corroborate vaccine hesitancy in Malawi. The first paper, by Chimatiro et al. [19], reports that COVID-19 vaccine hesitancy and refusal in Malawi are associated with myths. The myths are circulated in the community through social media sites such as WhatsApp and Facebook. Participants believed information that was being circulated on social media, such as that vaccines caused blood clots and infertility. In addition, religious leaders discouraged people from receiving the vaccine. Chimatiro et al. [19] uncovered that COVID-19 was perceived as a disease of wealthy people; others believed that it signaled the end of the world and the pandemic could not be cured. This negatively contributed to vaccine uptake.

The paper by Safary and Mtaita [20] looks into factors leading to hesitation and/or refusal to COVID-19 vaccination in Malawi. Fear of getting vaccinated due to potential reactions and side effects led to hesitancy of COVID-19 vaccination. The most perceived serious side effects reported are infertility and the potential formation of blood clots. According to Safary and Mtaita [20], the COVID-19 vaccine is perceived as dangerous and ineffective, thus one could still get the infection even after being fully vaccinated. Additionally, study participants questioned the long-term efficiency and efficacy of the vaccine. There are discussions of either having the COVID-19 vaccination on a yearly basis or adding additional booster doses. Such opinions deter respondents from getting vaccinated [20]. Some respondents felt that the vaccination rollout was part of a clinical trial hence did not want to participate in it. The authors advocate for measures to increase COVID-19 vaccine uptake and acceptance, targeting fear and misinformation.

Ao et al. [21] investigated vaccination rates for COVID-19 among Malawians. They also assessed the level of COVID-19 vaccine hesitancy among Malawians and explored the factors influencing vaccination and willingness to get vaccinated against COVID-19. The authors exposed that there is so much ignorance or incorrect knowledge about vaccines. This led to distrust and rejection of COVID-19 vaccines among Malawians. Ao et al. [21] recommend that individuals should be aware of existing health risks and take protective measures. The benefits of vaccination also need to be highlighted. Individuals should know that vaccines protect them from untimely deaths. Additionally, as recommended by Ao et al. [21], we can spread information on real-life COVID-19 cases and successful vaccination stories to promote vaccination behavior. We should also track and address rumors and misinformation about vaccines to rebuild public confidence in vaccination. At the same time, Malawi has its own unique cultural and religious background, so it is essential to work with trusted community leaders. Religious leaders can also act as vaccine advocates, using existing trust relationships to promote vaccination.

Furthermore, Aron et al. [22] describe attitudes toward the uptake of the COVID-19 vaccine and perceptions in patients with non-communicable diseases (NCDs) and their caregivers in rural Malawi. Vaccine uptake and intention to vaccinate among this group of vulnerable non-communicable disease patients and their caregivers in rural Malawi were three times higher than the general public’s current vaccination rate but substantially below the vaccination target of 70%. However, vaccines were perceived as unsafe or designed to harm and commonly associated with death, severe disability, infertility, and evil. Ojong and Agbe [23] exposed COVID-19 vaccine anxieties. Vaccine anxiety was not limited to any particular class or social status. According to Ojong and Agbe [23], the fact that a person was vaccinated did not indicate that they had no anxiety, thus context matters.

Holden et al. [24] exposed that some Malawians did not receive vaccines due to religious convictions and beliefs. They believed that getting the vaccine could result in receiving a triple-six (666) mark from the devil, a negative omen among devout Christians strongly associated with trust in the vaccine. Trust in vaccines had a strong impact on demand for vaccines and vaccine hesitancy. Furthermore, certain religious groups are associated with more of these pandemic-related beliefs, lower trust in vaccines and lower vaccine demand or stronger vaccine hesitancy [24]. Some of the Malawians belonging to the Seventh Day Adventists, the Pentecostals, and the community of Christ church have had significantly lower trust in the vaccine than those belonging to the Roman Catholic Church (base category) [24]. On the other hand, the authors found that trust in politicians, health personnel, and trust in general contributed to strengthening confidence in vaccines.

Furthermore, research has demonstrated that vaccination intention and behavior can be influenced. Age, education, religion, and occupation have a substantial impact on vaccination hesitancy rates against COVID-19 [25]. According to Mchawa et al. [25], participants in older age groups (40–49 years) and above 50 years are less likely to have vaccine hesitancy compared to younger adults aged 18–29 years. Probably, the older adults may perceive to be at a greater risk of severe illness and complications from COVID-19 and would be more motivated to get vaccinated [26]. Mchawa et al. [25] observed that people with a higher educational level might act better in understanding the knowledge of the vaccination importance of COVID-19 vaccine, so they performed a lower vaccine hesitancy. The vaccine hesitancy rate of Christians was dramatically higher than the Muslims [25]. Furthermore, individuals with lower monthly incomes have a higher acceptance of the COVID-19 vaccine [22]. This is widely believed due to government’s policy of free vaccines [22].

## Discussion

This paper aimed to investigate vaccine hesitancy among Malawians in the context of COVID-19 vaccination. The reviewed papers demonstrate that vaccine hesitancy in Malawi occurs as a result of myths and misinformation. The vaccines are viewed as dangerous or intended to cause harm, such as the myth that COVID-19 vaccine would cause infertility, severe disability, and death. There were also reports that COVID-19 vaccination is being considered experimental and ineffective; the anticipated and observed vaccine side effects led to hesitancy. The review revealed that some people did not receive the vaccine due to religious convictions and beliefs. They believed that receiving the vaccine could result in receiving a triple-six (666) mark from the devil. All these factors undermine the confidence that is crucial for sustaining collective immunity in vaccine programs. COVID-19 vaccine hesitancy has also been reported in some of the healthcare personnel [27]. Despite the fact that COVID-19 vaccines have been proven to be safe [28], some healthcare workers remain hesitant. It is not surprising that the vaccines that targeted persons at risk of acquiring the COVID-19 pandemic in Malawi such as healthcare workers, the elderly, and people with underlying comorbidities like diabetes and asthma got expired and discarded in May 2021 [9].

Although vaccination is framed as a collective duty in which citizens of welfare states contribute to population health as a measure of good citizenship [8], the right to autonomy should also be considered. Though public health programs are argued to limit freedom, a patient-centered approach is important for trust and decision-making. Therefore, efforts to address vaccine hesitancy should not rely on coercive public policies; instead, they should focus on citizen engagement to inform a patient-centered approach and cultivate an ethically consistent strategy [11] hence the need to strengthen the relationship between individual, collective, and institutional responsibility to prevent vaccine hesitancy and promote herd immunity. Additionally, it is indicated that the success of the COVID-19 vaccination program depends on the proportion of the population willing to be vaccinated [29]. Recent estimates suggest that up to 70% of the population may require vaccination to bring an end to the current pandemic [30], thus highlighting the need for a speedy rollout of vaccines to bring normalcy back as soon as possible.

In the pursuit of dealing with vaccine hesitancy, it has been earlier suggested that governments should communicate vaccine risks and benefits in a responsible manner and take responsibility for individuals negatively affected by the adverse effects of the vaccines [4]. Furthermore, vaccine skeptics should not be treated as ill-informed or less educated; their concerns should be addressed respectfully. In the event that individuals suffer from the side effects of vaccines, the public should get support from the government [4] to responsibly remedy concerns and restore trust. Finally, the citizens of Malawi need to be actively involved in the structure and modes of delivery of the COVID-19 vaccine. Additionally, the stakeholders responsible for vaccine rollout should acknowledge community efforts in vaccine acceptance and determine areas that require improvement to maintain vaccine acceptance [12].

Additionally, Chimatiro et al. [19] suggest that health specialists engage with religious leaders so that they can provide accurate messages to their followers. The vaccine uptake could be improved by effective community engagement by role models who advocate the vaccine and by integrating COVID-19 vaccination into community outreach services. False information related to COVID-19 vaccine may circulate through rural communities. Community engagement efforts should be directed at sensitizing the community on the need for vaccination against COVID-19 so that people should develop positive perceptions. Similarly, in Zambia, Pugliese-Garcia et al. [31] reported that people were hesitant to take the oral cholera vaccine because of religious beliefs. We cannot ascertain how the COVID-19 vaccine is associated with religious beliefs; however, we assume that inadequate and incorrect information could contribute to this line of thinking. Constant community sensitization would increase knowledge and awareness among individuals with little or no education and improve the uptake of health interventions [19, 20]. The article further argues that we live in a modern world where new technologies and social media platforms have resulted in a flood of publicly accessible information [16]. As recommended by Safary and Mtaita [20], interventions such as individual sensitization and motivational interviewing should be considered for guiding individuals toward considering COVID-19 vaccination. Religion plays a strong role in many African nations and many countries across the globe. Most of the religious congregations in Malawi and elsewhere belong to international networks that could potentially help protect against the pandemic though there may be persistent beliefs that are hard to change [24]. It may be important to address some of the religious groups and their leaders to promote vaccine demand, thereby reducing vaccine hesitancy.

### Limitations

This scoping review found 20 articles; however, only seven articles were used to analyze COVID-19 vaccination reluctance in Malawi due to limited studies on this topic. Additionally, COVID-19 vaccinations have been demonstrated to offer advantages that outweigh the disadvantages. However, there is little research that contextualizes this phenomenon in relation to vaccine hesitancy in Malawi. The review was limited to studies conducted in Malawi; as such the results may not be generalized to other settings/countries worldwide.

## Conclusions

Vaccine hesitancy is a growing problem that affects vaccine uptake. Vaccine hesitancy could derail global efforts to end the COVID-19 pandemic. Therefore, it is vital that the Malawi government and other stakeholders should understand why people are uncertain about receiving the COVID-19 vaccine. The government should also take action in order to reduce hesitation. Firstly, tackling vaccine hesitancy necessitates improved information communication between healthcare professionals and Malawians, including full disclosure of immunization information at the point of delivery. Secondly, misconceptions about the COVID-19 vaccination hinder the vaccine’s predicted success. As a result, response techniques to address misunderstandings must be implemented. All potential barriers to vaccine acceptance must be proactively addressed in a culturally and linguistically sensitive manner that includes the involvement of religious leaders.

### Supplementary Information

**Additional file 1.** Detailed search strategy and results

## Data Availability

All analyzed data are included in this article.
