# Supplementary material for: COVID-19 vaccination hesitancy among Malawians: a scoping review
Source: Syst Rev. 2024 Feb 28;13:77. doi: 10.1186/s13643-024-02499-z (PMC10900596; doi:10.1186/s13643-024-02499-z)
Supplement: Supplementary file 1 — Additional file 1. Detailed search strategy and results [file 13643_2024_2499_MOESM1_ESM.pdf]

Additional file

Covid-19 Vaccination Hesitancy among Malawians: A Scoping Review

Ellen Nkambule*[1], Chingatichifwe Mbakaya [2,3]

[1]St John's Institute for Health, Mzuzu, Malawi

[2]University of Livingstonia , Public Health, Mzuzu, Malawi

[3]Mzuzu University, Biological Sciences Department

On July 10th, 2023, all databases were searched as depicted in the table below. The phrase 'Malawi' was used to find studies conducted in this nation. We did not incorporate a date filter because we expected papers on COVID-19 to be released during the epidemic

Detailed search strategy and results

Table 1: Search terms

**Database**	**Terms used**	
**Results from 10th July,2023, 8:00am**		
CINAHL	vaccin* (hesita* OR negative perception* OR attitude OR sentiment OR distrust OR trust OR perspectives OR opinions*)) OR (attitude AND towards AND vaccines) OR (opinions AND on AND vaccines) OR (perspectives AND on AND vaccines) AND (coronavirus 2019) OR (covid 19) OR (covid-19) OR (coronavirus AND disease 2019) OR (2019 novel AND coronavirus) OR (2019-novel AND cov) OR (covid 2019) OR (2019 ncov) OR covid19 OR ncov-2019 OR ncov2019 OR (ncov 2019) OR (covid-19) OR (severe AND acute AND respiratory AND syndrome AND coronavirus 2) OR (2019-ncov) OR (sars-cov-2) OR novel OR coronavirus) 'SARS-CoV-2' OR 'SARS-2' OR 'severe acute respiratory syndrome coronavirus 2' AND Malawi	20
**Results from 10th July,2023, 11:00am**		
PubMed	(((vaccin* hesita* OR * negative attitude OR sentiment OR distrust OR trust *))) AND ((towards))) AND vaccines))) OR (negative opinions AND on AND vaccines) OR AND vaccines))) AND (((Malawi))	80
OVID Medline	((vaccin* adj8 (hesita* or accept* or perception* or attitude or sentiment or distrust or trust or perspectives or opinions*)) or attitude towards vaccines or opinions on vaccines or perspectives on vaccines) AND (coronavirus 2019 or COVID 19 or COVID-19 or coronavirus disease 2019 or 2019 novel coronavirus or 2019-novel CoV or COVID 2019 or 2019 ncov or COVID19 or nCoV-2019 or nCoV2019 or nCoV 2019 or COVID-19 or Severe acute respiratory syndrome coronavirus 2 or 2019-ncov or SARS-CoV-2 or novel novel coronavirus).	50
**Results from 10th July,2023, 6:00pm**		
Google scholar	COVID-19 AND vaccine hesitancy AND Malawi	99
